# Mass Spectrometry of Minimal Change Disease and Focal Segmental Glomerulosclerosis

**DOI:** 10.1016/j.ekir.2025.09.043

**Published:** 2025-10-11

**Authors:** Fernando C. Fervenza, Maria J. Vargas-Brochero, Hanna Debiec, Benjamin Madden, Luciana Andrade, Ladan Zand, Pierre Ronco, Sanjeev Sethi

**Affiliations:** 1 Division of Nephrology and Hypertension, Mayo Clinic, Rochester, Minnesota, USA; 2 Sorbonne Université, Paris, France and Institut National de la Santé et de la Recherche Médicale, Unité Mixte de Recherche S 1155, Paris, France; 3 Medical Genome Facility, Proteomics Core, Mayo Clinic, Rochester, Minnesota, USA; 4 Division of Nephrology, Centre Hospitalier du Mans, Le Mans, France; 5 Department of Laboratory Medicine and Pathology, Mayo Clinic, Rochester, Minnesota, USA

**Keywords:** focal segmental glomerulosclerosis, laser microdissection, mass spectrometry, minimal change disease

## Abstract

**Introduction:**

The relation between minimal change disease (MCD) and primary focal segmental glomerulosclerosis (FSGS) (pFSGS) remains debatable, and the recent discovery of antinephrin and antislit antibodies, supports the hypothesis of a continuum disease. However, the overall expression of podocyte proteins in these disease states is not known.

**Methods:**

In this observational proteomic study, we used laser microdissection and mass spectrometry (LMD/MS) to determine the proteomic profile of podocyte proteins in kidney biopsies of adult patients with MCD (*n* = 6), pFSGS (*n* = 7), secondary FSGS (sFSGS) (*n* = 10), and genetic FSGS (gFSGS) (*n* = 6). Time-zero kidney transplant (T0) (*n* = 6), IgA nephropathy (IgAN) (*n* = 5) and membranous nephropathy (MN) (*n* = 8) served as nonproteinuric and proteinuric controls, respectively. MS results were expressed as total spectrum representing the relative abundance of the specific protein. In addition, immunofluorescence (IF) staining assessing nephrin (NPHS1) and podocin (NPHS2) were performed.

**Results:**

LMD/MS show moderate baseline total spectrum of podocyte proteins, NPHS1, NPHS2, CD2-associated protein, alpha-actinin-2, inverted formin-2 (INF2), and dystroglycan 1 (DAG1) in T0 and MN controls. In contrast, there was a significant loss of all 6 podocyte proteins in MCD, pFSGS, sFSGS, and gFSGS but not in MN cases. IF staining confirmed podocyte loss of NPHS1 and NPHS2 in MCD, pFSGS, sFSGS, and gFSGS but not in T0 or MN.

**Conclusion:**

LMD/MS and IF staining show significant and comparable loss of podocyte proteins involving the slit diaphragm and actin cytoskeleton in MCD, pFSGS, sFSGS, and gFSGS suggesting a common final pathway of podocyte injury. Surprisingly, despite similar degrees of proteinuria, MN was not associated with significant loss of podocyte proteins.

## Graphical abstract

**Figure:**
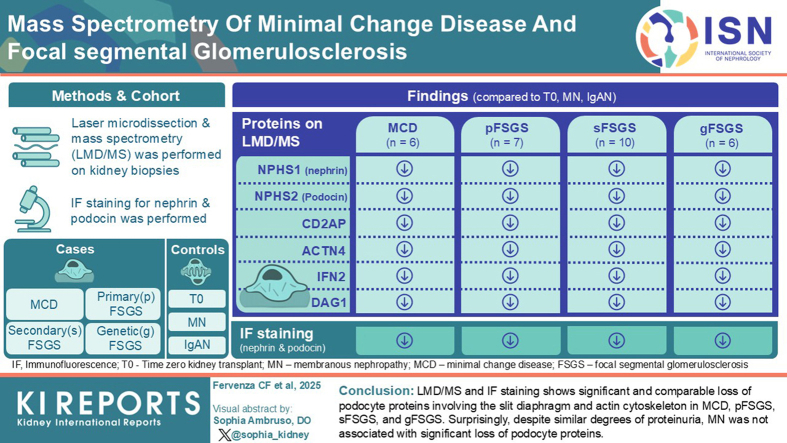

MCD and presumed pFSGS are common causes of nephrotic syndrome (NS). On kidney biopsy, MCD is characterized by normal-appearing glomeruli on light microscopy (LM), negative IF studies, and widespread foot process effacement (FPE) on electron microscopy (EM). In contrast, pFSGS is characterized by the presence of segmental sclerosis, negative IF, and widespread FPE on EM.^1^^,^^2^ Both lesions are indistinguishable on EM. Clinically, the majority of patients with MCD achieve complete remission of proteinuria following immunosuppression therapy, whereas a significant number of patients with pFSGS are resistant to immunosuppressive therapy and may eventually progress to end-stage kidney disease. Both MCD and pFSGS are prototypical examples of primary podocytopathies secondary to a circulating factor(s) that is or are toxic to the podocyte. Recently, antinephrin antibodies have been reported in MCD and a subset of patients with pFSGS.^3^ Whether MCD and pFSGS, are part of the spectrum of the same disease, has been debated.^4^ Support for this hypothesis comes from cases that were originally diagnosed as MCD on the initial kidney biopsy, and subsequent kidney biopsies showed an FSGS lesion.^5^ Similarly, kidney biopsies of patients with recurrent pFSGS posttransplant are initially characterized by normal glomeruli on LM, negative IF, with widespread FPE on EM.^6^ With time, however, FSGS lesions develop in subsequent allograft kidney biopsies. In contrast, sFSGS result from a number of different etiologies, including obesity, unilateral renal agenesis, drugs, and viral infections; and gFSGS is defined as the presence of FSGS with an underlying genetic cause identified by genetic analysis. From the clinical stand point, sFSGS often presents with gradual increase in proteinuria over time without development of NS, and pathology shows FSGS lesions with varying degrees of FPE on EM.

It is hypothesized that the podocyte is the primary site of injury in MCD and pFSGS, whereas podocyte injury is secondary, for example, glomerular hypertension or hyperfiltration, in sFSGS. In this study, we aimed to study the proteomic profile of podocyte proteins using LMD/MS of glomeruli from MCD, pFSGS, sFSGS, and gFSGS. We hypothesized that MS/MS could distinguish these 4 entities.

## Methods

### Patient Selection

Biopsies of patients aged > 18 years received in the Renal Pathology Laboratory, Department of Laboratory Medicine and Pathology, Mayo Clinic, were evaluated for diagnosis and interpretation. Patients with MCD were defined by the presence of NS (i.e., proteinuria > 3.5 g/24 h and serum albumin < 3.5 g/dl), normal kidney biopsy on LM, negative IF, and widespread (> 80%) FPE on EM examination, and complete remission of proteinuria following immunosuppression therapy. pFSGS was defined as the presence of NS, with an FSGS lesion on kidney biopsy on LM, negative IF, and widespread FPE on EM, negative genetic analysis, and irrespective of response to immunosuppressive therapy. sFSGS was defined by the presence for an FSGS lesion on LM, negative IF, segmental FPE (< 80%), absence of NS and no genetic cause. gFSGS was defined by a clinical and pathological pattern consistent with FSGS in the context of a positive genetic analysis identifying pathogenic or likely pathogenic variants in podocyte-related genes. Cases of FSGS of undetermined cause were excluded. Kidney biopsies of 6 cases of MCD, 7 of pFSGS, 10 of sFSGS, and 6 of gFSGS, were included in the study. The gFSGS cases included mutations in the following genes: *COL4A3/COL4A4* (1 case), *COL4A3* (1 case), *COL4A4* (1 case), *COL4A5* (1 case), and *INF2* (2 cases). Proteomic profile of the podocyte proteins living donor kidney transplants preimplantation (time 0) (*n* = 6) served as nonproteinuric controls, IgAN kidney biopsies (*n* = 5) without signs of chronic scarring (no tubular atrophy or interstitial fibrosis) and MN kidney biopsies (*n* = 8) served as proteinuric controls.

Data were collected via manual chart review, and the patient’s demographic and clinical characteristics were collected from medical records. The degree of FPE was categorized as mild (40%), moderate (40%–79%), and severe (> 80%) based on previously descriptive terminology.^7^ The study was approved by the Mayo Clinic Institutional Review Board. In this study, sex refers to biological attributes (male or female) as documented in clinical records. Gender was not assessed and is not reported. All analyses use sex as a biological variable. This approach aligns with the journal’s guidance and the SAGER recommendations.

### LMD/MS

Protein identification by laser capture microdissection, trypsin digestion, nano-liquid chromatography orbitrap MS/MS was performed as previously described.^8^ Briefly, 3 to 4 sections of 10-micron thick formalin-fixed paraffin embedded sections that were mounted on polyethylene naphthalate membrane glass slides then deparaffinized in xylene and rehydrated. The glomeruli were microdissected free of the Bowman’s capsule using a Zeiss Palm Microbeam microscope to accumulate approximately 300000 to 500000 mM^2^ per case into the cap of a 0.5 ml tube containing 35 μl of 100 mM Tris pH 8.2 / 0.005% zwittergent. The number of glomeruli dissected were counted per case. Because the polyethylene naphthalate glass slide contains 3 to 4 sections of the renal biopsy core(s), the same glomerulus may be cut 3 to 4 times. In other words, a core containing only 5 to 10 glomeruli may yield > 20 glomeruli on dissection. The average areas dissected for T0, MN, MCD, pFSGS, sFSGS, and gFSGS were 449847, 348600, 445085, 387249, 468939, 446802 mM^2^, respectively. Only nonglobally sclerosed glomeruli were dissected. Each case was processed separately and the dissected fragments from individual cases were not pooled. The collected formalin-fixed paraffin embedded fragments were centrifuged and heated at 98 °C for 60 minutes for protein extraction, then reduced with tris carboxyethyl phosphine, alkylated with iodoacetamide, followed by digestion with trypsin/LysC mix (Promega) at 37 °C overnight. The digest mixture was acidified with dilute trifluoroacetic acid and loaded onto a C18 trap column (EXP Halo 2.7 um, Optimize Technologies). The peptides were separated using a 100 μm × 40 cm C18 column (PepSep 1.5 um; Bruker) running an 80-minute gradient with 0.1% formic acid / acetonitrile buffers on a Thermo Ultimate 3000 RSLCnano HPLC system coupled to a Thermo Scientific Exploris480 or Qexactive Orbitrap Mass Spectrometer (Thermo Fisher Scientific, Bremen, Germany) set up for data-dependent acquisition nano-flow liquid chromatography electrospray tandem MS/MS (nanoLC-ESI-MS/MS) analysis. The MS raw data files were searched with Andromeda against the Swissprot human database (ver. 2023_01) plus a reverse decoy database in MaxQuant (ver1.6.17), setup for MS1 peak intensity-based quantification. The matched peptides and proteins were filtered at a 1% false discovery rate and the MaxQuant reported protein level intensity-based absolute quantitation (iBAQ) values are used for comparisons between the 2 groups using an inhouse R-script to calculate fold-change and *P*-values.^9^^,^^10^ The protein of interest was compared with actin, vimentin, laminin, and collagen IV to ensure equal amounts of baseline proteins in the sample. Sample groups missing 50% of values were removed for comparison with no imputation applied. Samples were normalized by median subtraction and *t* test comparisons made with protein iBAQ value log2 ratios. The MS proteomics data have been deposited to the ProteomeXchange Consortium via the PRIDE partner repository with the dataset identifier PXD057165 and 10.6019/PXD057165.^11^

### IF Staining for NPHS1 and NPHS2

IF staining was performed on formalin-fixed paraffin embedded sections retrieved for 30 minutes with sodium citrate buffer of pH 6 in pressure cooker equipment. The NPHS1 primary antibody (rabbit monoclonal ab 216341 abcam) and NPHS2 primary antibody (rabbit monoclonal ab183703 abcam) were diluted to 1:200 in blocking solution (2% calf fetal serum and 2% normal goat serum) and incubated overnight at 4 °C with retrieved biopsy sections. After washing, the slides were incubated with secondary antibody Alexa Fluor 488-conjugated Fab fragment goat anti-rabbit IgG (Life technologies, Thermo Fisher Scientific). Finally, slides were placed in mounting medium (Thermo Fisher Scientific) and covered with LDS2460EP cover glasses. Stained sections were evaluated with Axio Examiner Z1 Carl Zeiss microscope.

#### Statistical Methods

For the analysis, we used MS/MS count as a continuous variable, which we summarized using mean (SD) for normally distributed data and median interquartile range (IQR) for nonnormally distributed data. For comparing continuous variables between 2 groups, *t* test or the Mann–Whitney *U* test was used, depending on the distribution of the data. For comparisons involving > 2 groups, 1-way analysis of variance was applied to normally distributed variables, whereas the Kruskal-Wallis test was used for nonnormally distributed variables. When the Kruskal-Wallis test indicated significant differences, pairwise comparisons were performed using the Mann-Whitney *U* test with Bonferroni correction to adjust for multiple testing. All statistical analyses were performed per protein, and results were visualized using bar plots with error bars to represent variability across groups. A heatmap of Bonferroni-corrected *P*-values was generated to visualize the pairwise comparisons among groups, with color intensity representing the magnitude of the corrected *P*-values.

*P*-values < 0.05 were considered statistically significant. All statistical analyses were performed using BlueSky Statistics software v. 10.3 (BlueSky Statistics LLC, Chicago, IL).

## Results

### Volcano and Cluster analysis of MCD, pFSGS and sFSGS

**Figure 1:**
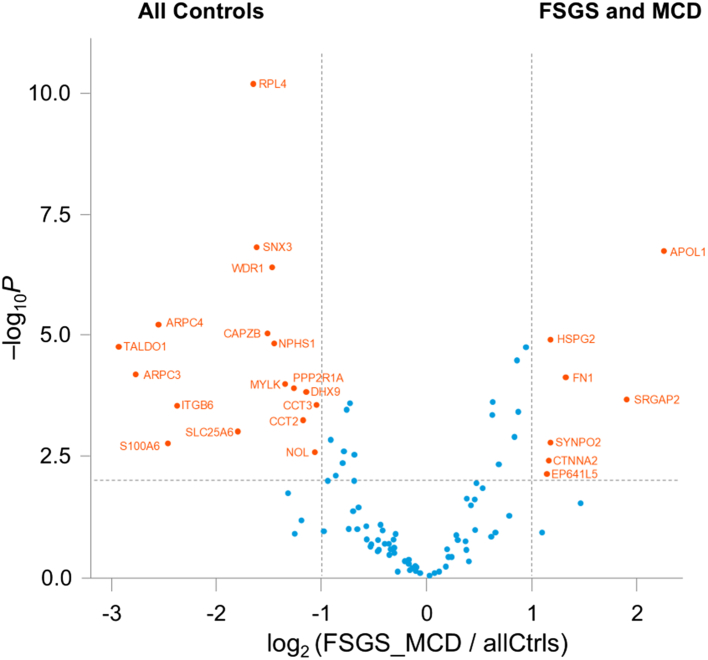
Volcano plot: The mean log2 iBAQ protein values for time-zero kidney transplant and membranous nephropathy (all controls) and MCD, pFSGS and sFSGS (MCD and FSGS) are compared and displayed in a volcano plot where the proteins highlighted in red indicate differences ≥ 2-fold change with a −log10 *P*-values ≥ 2. Proteins that are higher in MCD and FSGS cases are in the upper right, whereas proteins higher in all control’s cases are in the upper left. FSGS, focal segmental glomerulosclerosis; iBAQ, intensity-based absolute quantitation; MCD, minimal change disease; pFSGS, primary FSGS, sFSGS, secondary FSGS.

The mean log2 iBAQ protein values show significant differences in the protein profile between T0 biopsies and MN cases compared with MCD, pFSGS, sFSGS, and gFSGS. The proteins that were increased in MCD, pFSGS, and sFSGS with > 2-fold change were the following: apolipoprotein L1, heparan sulfate proteoglycan 2, fibronectin 1, SRGAP2, synaptopodin 2, and catenin alpha 2 (Figure 1). Apolipoprotein L1 was the most significantly increased protein in this group when compared with T0 biopsies and MN cases. In contrast, compared with T0 and MN, there was generalized loss of proteomic signal for NPHS1, NPHS2, alpha-actinin-4, INF2, and DAG1 in MCD, pFSGS, sFSGS, and gFSGS. Compared with each other, only fibronectin 1 and heparan sulfate proteoglycan 2 showed > 2-fold increase in mean log2 iBAQ protein values in pFSGS compared with MCD (Supplementary Figure S1). There was a loss of multiple other proteins (data not shown). Principal component analyses (cluster analysis) showed tight clustering of the MCD, pFSGS, and sFSGS cases compared with T0 biopsies and MN cases (Supplementary Figure S2).

### LMD/MS Analysis of Podocyte Proteins in MCD, pFSGS, sFSGS, and gFSGS Compared With T0s and MN

**Figure 2:**
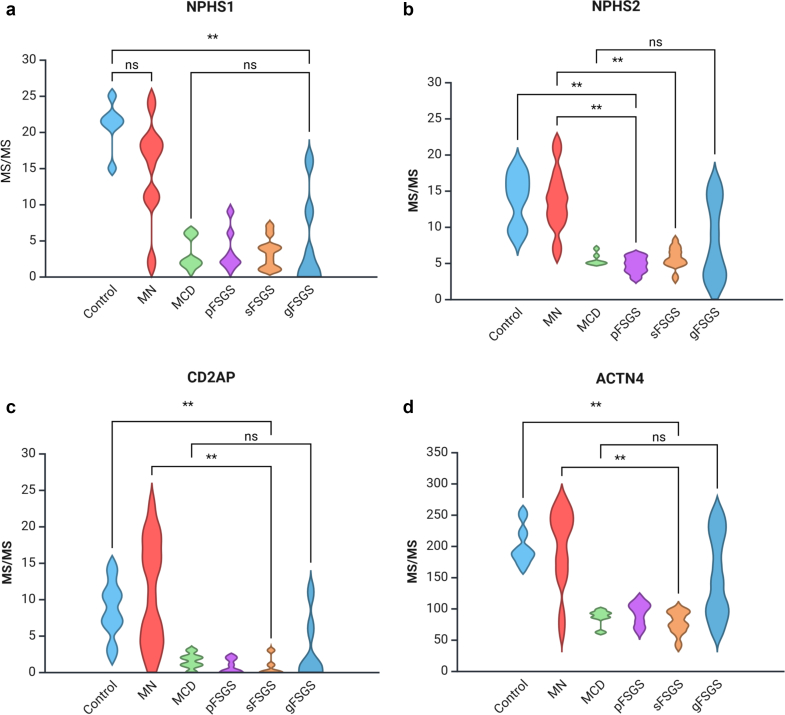

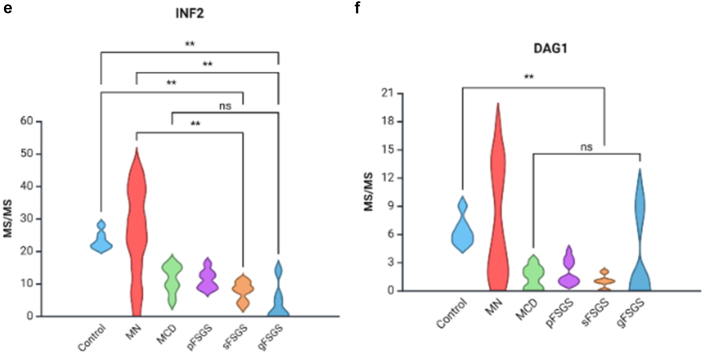
MS/MS values of all the podocyte proteins across different glomerular disease groups (control, MN, MCD, pFSGS, sFSGS, and gFSGS). Statistical significance is indicated as follows: *P* < 0.05 (∗∗), not significant (ns). (a) Nephrin (NPHS1), (b) podocin (NPHS2), (c) CD2-associated protein (CD2AP), (d) alpha-actinin-4 (ACTN4), (e) inverted formin-2 (INF2), and (f) dystroglycan 1 (DAG1). gFSGS, genetic focal segmental glomerulosclerosis; MCD, minimal change disease; MN, membranous nephropathy; pFSGS, primary focal segmental glomerulosclerosis, sFSGS, secondary focal segmental glomerulosclerosis.

**Figure 3:**
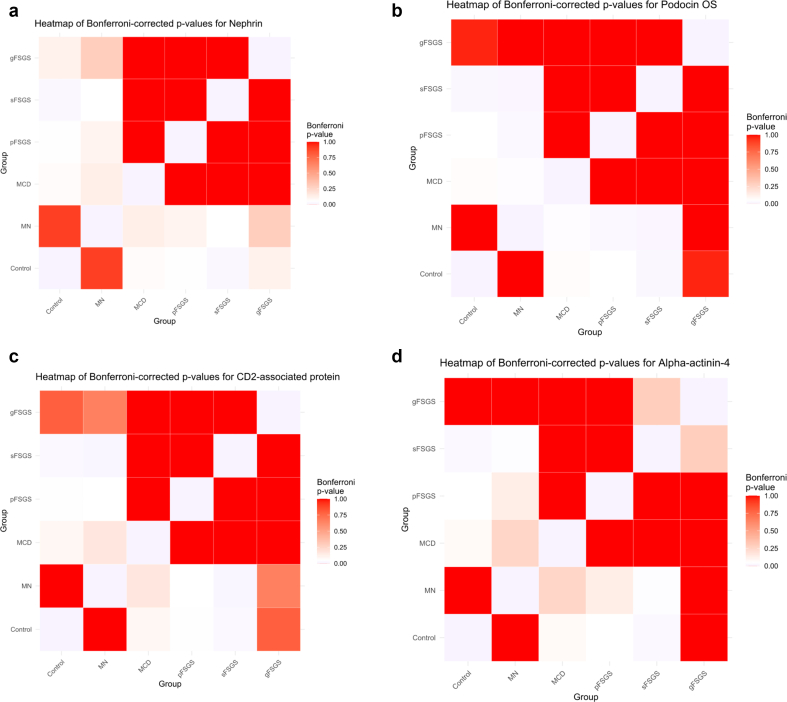

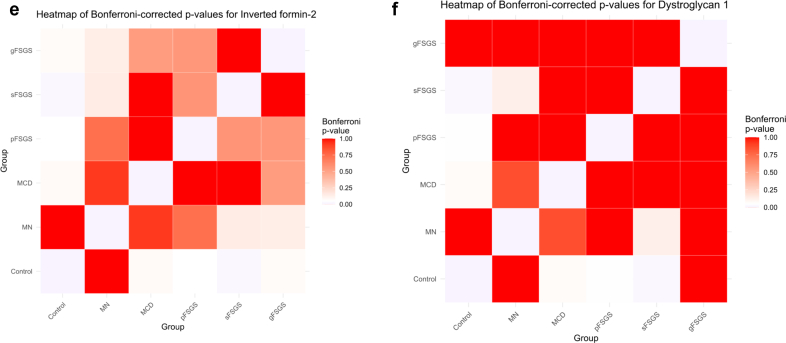
Heatmap of Bonferroni-corrected *P*-values for all podocyte proteins. Pairwise comparisons among control, MN, MCD, pFSGS, sFSGS, and gFSGS are shown. Color intensity reflects Bonferroni-adjusted *P*-values (white = low, red = high). (a) Nephrin (NPHS1), (b) podocin (NPHS2), (c) CD2-associated protein (CD2AP), (d) alpha-actinin-4 (ACTN4), (e) inverted formin-2 (INF2), and (f) dystroglycan 1 (DAG1). gFSGS, genetic focal segmental glomerulosclerosis; MCD, minimal change disease; MN, membranous nephropathy; pFSGS, primary focal segmental glomerulosclerosis, sFSGS, secondary focal segmental glomerulosclerosis.

We evaluated the spectral counts and sample-normalized iBAQ values of 6 major podocyte proteins that were identified in MCD (*n* = 6), pFSGS (*n* = 7), sFSGS (*n* = 10), gFSGS (*n* = 6), MN (*n* = 8), and T0 transplant (*n* = 6) groups and observed significant decreases in NPHS1, NPHS2, CD2-associated protein, alpha-actinin-2, INF2, and DAG1 in all 4 groups compared with MN and T0 control group (Table 1). In the analysis of protein expression across glomerular disease subtypes, MCD, pFSGS, and sFSGS exhibit similarly diminished MS/MS values, indicating a consistent reduction in MS/MS count levels among these conditions. In contrast, MN and gFSGS display markedly higher variability, with a broader distribution of values suggesting heterogeneous expression (Figure 2). In Table 1, we highlight the mean or median spectral count differences; and in Table 2, we illustrate the percent decrease in mean iBAQ values of the indicated comparisons. IgAN cases exhibited MS/MS values consistently closer to those observed in MN, and both conditions showed profiles more similar to the nonproteinuric controls (Table 1). This pattern suggests a relatively preserved podocyte protein profile in IgAN and MN, in contrast to the marked reductions seen in podocytopathies such as MCD, pFSGS, sFSGS, and gFSGS. Pairwise comparisons were performed using the Mann-Whitney *U* test with Bonferroni correction. NPHS1 was significantly reduced in all podocytopathy groups compared with controls, with no significant differences among the disease groups themselves. NPHS2 expression was markedly decreased in MCD, pFSGS, and sFSGS, but was relatively preserved in gFSGS. A similar pattern was observed for CD2-associated protein, alpha-actinin-2, and DAG1. INF2 expression, like NPHS1, were significantly reduced across all disease groups. Notably, 2 patients in the gFSGS group carried known *INF2* mutations, which may contribute to the observed reduction (Figures 2 and 3).

**Table 1:** Comparison of MS/MS spectral counts among control, MN, IgAN, MCD, pFSGS, sFSGS, and gFSGS groups

MS/MS counts									
Proteins descriptions	Gene	Control *n* = 6	MN *n* = 8	IgAN *n* = 5	MCD *n* = 6	pFSGS *n* = 7	sFSGS *n* = 10	gFSGS *n* = 6	*P*-value^a^
Nephrin	*NPHS1*	21.5 [21–22]	17 [11–18.2]	19 [16–20]	2 [2–5]	2.0 [2–4.5]	3.5 [1.2–4]	4.8 (6.43)	< 0.001
Podocin	*NPHS2*	14.5 [11–16.5]	13.5 [11–15.5]	9 [9–10]	5 [5–5.75]	5.0 [4.0–5.5]	5.5 [5–6.8]	6.5 (4.0–2.7)	< 0.001
CD2-associated protein	*CD2AP*	8.5 [7–10.8]	10.0 [4.8–17]	10 [7–10]	1.5 [1.0–2.0]	0 [0–1.5]	0 [0–1]	1 [0.5–4.75]	< 0.001
Alpha-actinin-4	*ACTN4*	200.5 (29.9)	200 (62.7)	248.2 (39.1)	86.17 (12.7)	92.71 (18)	77.4 (18.2)	152 (67.8)	< 0.001
Inverted formin-2	*INF2*	22.50 [21.25–24.5]	26 [17.7–35.5]	33 [32–34]	12 [9.2–14.7]	11 [9–13]	8 [5.7–9.7]	1.5 [0–5.3]	< 0.001
Dystroglycan 1	*DAG1*	6.0 [5.2–6.8]	5.5 [2.0–12.2]	9 [9–10]	1.5 [0.2–2.0]	1 [1–3]	1 [0–1]	0.5 [0–6.3]	< 0.001

gFSGS, genetic focal segmental glomerulosclerosis; IgAN, IgA nephropathy; IQR, interquartile range; MN, membranous nephropathy; MCD, minimal change disease; pFSGS, primary focal segmental glomerulosclerosis; sFSGS, secondary focal segmental glomerulosclerosis.

Data are presented as mean (SD) or median [IQR].

a *P* < 0.05 is considered significant (analysis of variance for continuous variables normally distributed and Kruskal–Wallis’s test for continuous variables with skewed distribution).

**Table 2:** Comparison of sample-normalized mean iBAQ values of MCD, pFSGS, and sFSGS groups to time 0 transplant controls and MN

Percent decrease in mean iBAQ values							
Proteins descriptions	Genes	MCD vs. Time 0 controls	MCD vs. MN	pFSGS vs. time 0 controls	pFSGS vs. MN	sFSGS vs. time 0 controls	sFSGS vs. MN
Nephrin	*NPHS1*	−92%	−84%	−91%	−83%	−97%	−94%
Podocin	*NPHS2*	−47%	−45%	−59%	−57%	−36%	−33%
CD2-associated protein	*CD2AP*	−99%	−100%	−100%	−100%	−100%	−100%
Alpha-actinin-4	*ACTN4*	−58%	−51%	−56%	−49%	−64%	−58%
Inverted formin-2	*INF2*	−59%	−48%	−47%	−34%	−56%	−45%
Dystroglycan 1	*DAG1*	−99%	−93%	−20%	−80%	−98%	−92%

iBAQ, intensity-based absolute quantitation; MCD, minimal change disease; MN, membranous nephropathy; pFSGS, primary focal segmental glomerulosclerosis; sFSGS, Secondary focal segmental glomerulosclerosis

Percent decrease determined using the normalized mean iBAQ values for MCS, pFSGS, and sFSGS groups compared with time 0 transplant controls and MN.

### IF Staining Results

Fourteen cases were selected for evaluation by IF staining for NPHS1 and NPHS2. These cases were chosen based on the availability of sufficient extra tissue for additional analysis. The selected cases included 3 patients with MCD, 4 with pFSGS, 3 with sFSGS, and 4 with gFSGS. These were analyzed alongside control cases, which included time-zero biopsies and cases of MN, IgAN, and lupus nephritis. Case selection was performed before the staining procedure, and the evaluator was blinded to the diagnosis at the time of assessment to reduce bias.

**Figure 4:**
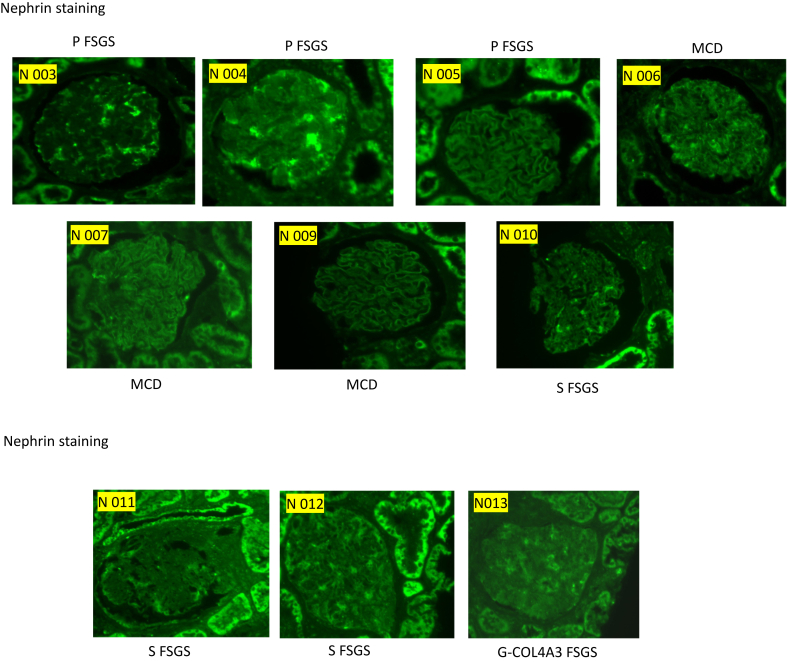

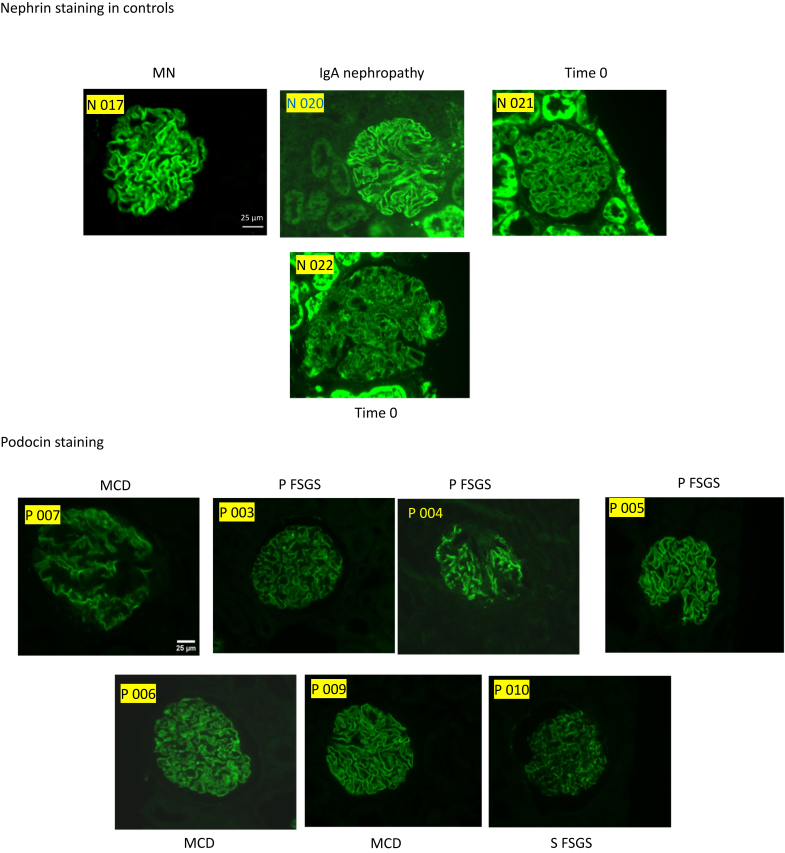

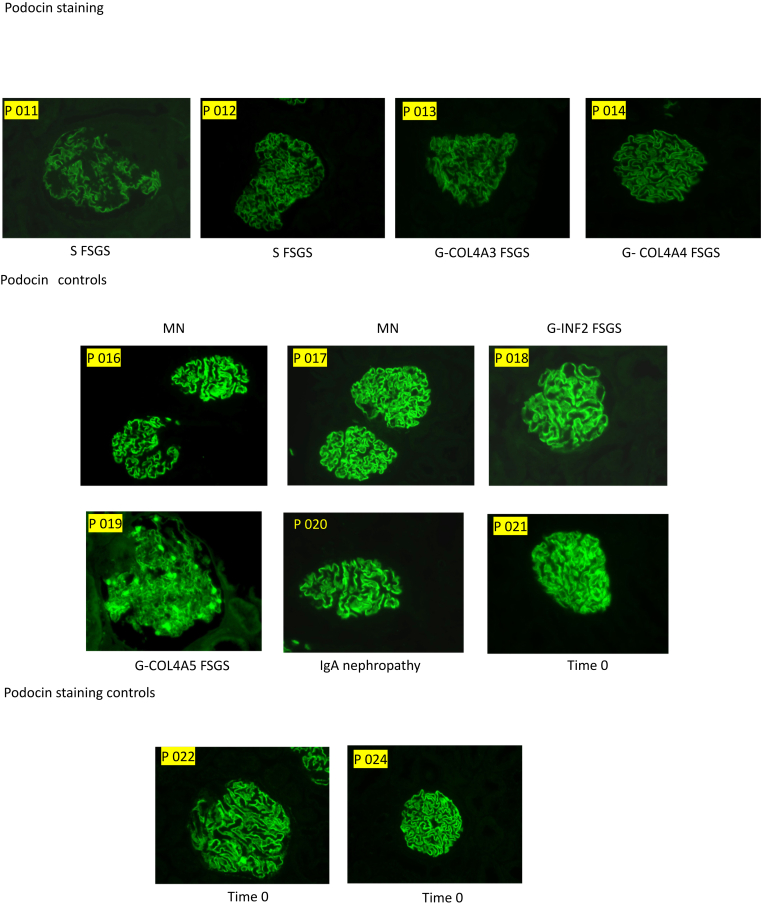
IF staining showed podocyte loss of nephrin and podocin in disease but not in controls, Bar = 25 μm. FSGS, focal segmental glomerulosclerosis; MCD, minimal change disease.

IF staining showed podocyte loss of NPHS1 and NPHS2 in MCD, pFSGS, sFSGS, but not in T0 or MN. Interestingly, gFSGS cases showed variable IF findings, with less NPHS2 loss observed in 2 genetic cases—P018 (*INF2* mutation) and P019 (*COL4A5* mutation). In addition, on closer analysis, one could discern 2 patterns of NPHS1 in the diseased conditions (MCD/FSGS) as follows: 1 patchy and 1 very weak, whereas NPHS2 showed a more homogeneously decreased to very weak or absent staining (Figure 4).

### Clinical Features of MCD, pFSGS, sFSGS, gFSGS, and MN

The median (IQR) age of the 37 patients was 48 (26,61) years, 22 (59.5%) were classified by biological sex as male. The median serum creatinine at the time of kidney biopsy was 1.20 (IQR: 0.88–1.70) mg/dl. Median proteinuria was 6.0 (IQR: 3.60–9.15) g/24 h, and the median serum albumin level was 3.05 (IQR: 2.00–3.80) g/dl. There were no significant differences in age, sex, or serum creatinine among the diagnostic groups. However, proteinuria levels were significantly higher in the MCD, pFSGS, and MN groups than in the gFSGS and sFSGS groups (*P* = 0.02), with correspondingly lower serum albumin levels in the same groups (*P* < 0.001) (Table 3).

**Table 3:** Clinical Findings in MCD, pFSGS, sFSGS, gFSGS, and MN cases

Patient number	Diagnosis	Age/sex	Serum Creatinine (mg/dl)	Proteinuria (g/24 h)	Serum Albumin (g/dl)	Light microscopy findings	EM findings
1	MCD	23/M	1.03	9.2	1.4	No GS No TA/IF	Diffuse FPE
2	MCD	47/M	4.4	10.6	1.6	No GS No TA/IF Acute tubular injury	Diffuse FPE
3	MCD	51/F	0.74	6.0	1.7	No GS No TA/IF	Diffuse FPE
4	MCD	21/F	0.5	5.3	2	No GS No TA/IF	Diffuse FPE
5	MCD	61/F	1.24	9.1	1.2	No GS No TA/IF Acute tubular injury	Diffuse FPE
6	MCD	54/M	1.3	6.2	2	No GS No TA/IF Acute tubular injury	Diffuse FPE
7	pFSGS	80/M	1.7	6.0	2.24	NOS FSGS Foam cells 27% GS No TA/IF	Diffuse FPE
8	pFSGS	66/M	2.1	14.2	2.7	NOS FSGS 10% GS Mild TA/IF	Diffuse FPE
9	pFSGS	23/M	1.2	23.1	2.7	NOS FSGS Foam cells 8.3% GS Mild TA/IF	Diffuse FPE
10	pFSGS	28/M	0.8	10.0	3.4	NOS FSGS 5% GS Mild TA/IF	Diffuse FPE
11	pFSGS	25/M	3.2	6.2	1.7	Collapsing FSGS No GS Mild TA/IF	Diffuse FPE
12	pFSGS	48/M	2.2	8.2	2.5	NOS FSGS 45% GS Moderate TA/IF	Diffuse FPE
13	pFSGS	41/F	0.9	4.0	2.9	Tip FSGS 7.4% GS Mild TA/IF	Diffuse FPE
14	sFSGS	35/F	1.7	3.9	3.8	NOS FSGS 9% GS Mild TA/IF Diffuse tubular dilatation	Moderate FPE
15	sFSGS	58/M	1.1	3.3	3.8	NOS FSGS 40% GS Mild TA/IF Glomerulomegaly	Moderate FPE
16	sFSGS	67/M	1.8	2.1	3.2	NOS FSGS 40% GS Moderate TA/IF Acute tubular injury	Mild FPE
17	sFSGS	54/M	1.3	5.8	4.8	Tip FSGS No GS Mild TA/IF	Mild FPE
18	sFSGS	19/M	1.2	2.9	4.2	Perihilar FSGS 16% GS Mild TA/IF	Mild FPE
19	sFSGS	71/M	1.5	6.0	3.7	NOS FSGS No GS Mild TA/IF Glomerulomegaly	Moderate FPE
20	sFSGS	21/M	1.4	1.8	3.5	NOS FSGS No GS Mild TA/IF Glomerulomegaly	Mild FPE
21	sFSGS	21/M	0.9	2.2	3.6	NOS FSGS No GS Mild TA/IF Glomerulomegaly	Mild FPE
22	sFSGS	70/M	2.5	4.5	3.9	NOS FSGS 57% GS Moderate TA/IF Glomerulomegaly	Mild FPE
23	sFSGS	35/F	0.7	2.2	4.1	NOS FSGS No GS Mild TA/IF	Moderate FPE
24	gFSGS COL4A3 and COL4A4	52/M	1.79	8.8	4.2	NOS FSGS 5% GS Mild TA/IF Glomerulomegaly	GMB segmental thinning no lamellations Mild FPE
25	gFSGS COL4A4	53/F	1.1	3.5	4.2	NOS FSGS 33% GS Mild TA/IF Glomerulomegaly	Moderate FPE
26	gFSGS INF2	26/F	0.8	5.7	4.3	NOS FSGS 5.2% GS No TA/IF	Diffuse FPE
27	gFSGS COL4A5	51/F	1.5	1.8	3.5	NOS FSGS 63% GS Moderate TA/IF	Moderate FPE
28	gFSGS INF2	19/M	1.3	7.0	4.0	NOS FSGS 43% GS Moderate TA/IF	Moderate FPE
29	gFSGS COL4A3	48/M	1.01	1.8	3.8	NOS FSGS 23% GS No TA/IF	GMB segmental thinning no lamellations Mild FPE
30	MN	66/F	1.2	11.0	1.6	Thickening of GBM Mild TA/IF	Subepithelial electron-dense deposits Segmental FPE
31	MN	63/F	1.07	3.7	NA	Thickening of GBM Mild TA/IF	Subepithelial electron-dense deposits Diffuse FPE
32	MN	71/F	4.0	NA	NA	Thickening of GBM, with spikes and craters Severe IFTA	Subepithelial electron-dense deposits Diffuse FPE
33	MN	66/F	NA	6.0	1.8	Thickening of GBM Mild TA/IF	Subepithelial electron-dense deposits Mild FPE
34	MN	29/M	0.84	9.8	2.0	Thickening of GBM, with spikes and craters No TA/IF	Subepithelial electron-dense deposits Diffuse FPE
35	MN	19/F	0.6	NA	NA	Thickening of GBM No TA/IF	Subepithelial electron-dense deposits Diffuse FPE
36	MN	30/M	0.7	12.0	2.1	Thickening of GBM, with spikes and craters Mild TA/IF	Subepithelial electron-dense deposits Diffuse FPE
37	MN	55/F	0.7	11.5	2.4	Thickening of GBM Mild TA/IF	Subepithelial electron-dense deposits Diffuse FPE

EM, electron microscopy; F, female; FPE, foot process effacement; FSGS, focal segmental glomerulosclerosis; gFSGS, genetic FSGS; GBM, glomerular basement membrane; GS, glomerulosclerosis; INF2, inverted formin-2; M, male; MCD, minimal change disease; MN, membranous nephropathy; NA, not available; NOS, not otherwise specified; pFSGS, primary FSGS; sFSGS, secondary FSGS; TA/IF, tubular atrophy and interstitial fibrosis.

Mild FPE: <25%, moderate FPE: 25%–80%, diffuse FPE: > 80%.

*COL4A3, COL4A4, COL4A5*, Genes encoding type IV collagen α3, α4, and α5 chains.

### Biopsy Findings

All MCD biopsies showed diffuse FPE, with no evidence of glomerulosclerosis, tubular atrophy, or interstitial fibrosis. Two cases exhibited associated acute tubular injury. All patients were receiving steroid therapy and achieved complete proteinuria remission at the last follow-up.

In the pFSGS group, all cases had diffused FPE, consistent with diagnostic criteria. The median percentage of glomerulosclerosis was 8.30% (IQR: 6.20%–18.50%), with mild tubular atrophy or interstitial fibrosis observed. All patients initiated immunosuppressive therapy following biopsy. Among the 7 patients, 6 achieved either complete or partial proteinuria remission; 1 patient was lost to follow-up.

Among the 10 sFSGS cases, 6 (60%) had mild FPE, whereas the remaining showed moderate effacement. Mild tubular atrophy or interstitial fibrosis was present in 8 patients (80%). Glomerulomegaly was observed in 50% of cases on LM, and the median percentage of glomerulosclerosis was 4.5% (IQR: 0%–34%). Two patients received immunosuppressive therapy before kidney biopsy without clinical response. One of these patients underwent genetic testing during pretransplant evaluation, which was negative.

In the gFSGS group, 4 patients had abnormalities in collagen genes associated with the Alport spectrum, whereas the remaining had mutations in the INF2 gene. Two patients presented with thinning of the glomerular basement membrane, and 1 case showed diffuse FPE, with the rest exhibiting moderate or mild effacement. The median percentage of glomerulosclerosis was 28% (IQR: 9.65%–40.5%).

## Discussion

MCD and pFSGS are classical examples of primary podocytopathies. It is hypothesized that in MCD, the pathogenic process is abrogated using immunosuppression with recovery of the podocytes. In contrast, persistence of pathogenic process results in podocyte death and depletion leading to areas of denuded glomerular basement membrane with subsequent development of focal sclerosis in an attempt to heal the areas of denuded glomerular basement membrane. However, do they represent an extreme spectrum of the same pathogenic process? The recent discovery of antinephrin antibodies^3^^,^^12^ and antislit diaphragm atibodies^13^^,^^14^ in a large number of patients with MCD and smaller number with pFSGS suggests overlap of these diseases in at least some patients. In the case of antinephrin-mediated disease, the explanation why some patients diagnosed with MCD do not progress to FSGS may depend on the antibody levels, disappearance of antibodies following immunosuppressive therapy, or other unknown factors.^15^^,^^16^ In contrast, sFSGS results from a variety of causes that are independent of a “circulating permeability factor,” that results in a heterogeneous pattern of injury, as evidenced by variable degrees FPE on EM.^12^^,^^17^

The aim of the study was to use MS/MS data to answer the question: are MCD and pFSGS different diseases or a spectrum of the same? The answer to our initial hypothesis was that MS/MS could not differentiate MCD from pFSGS, sFSGS, and gFSGS. These findings contrast with results obtained in biopsies of patients with MN, where we expected significant loss of podocyte proteins because there is extensive FPE in MN secondary to immune complex accumulation in the subepithelial region of the glomerular basement membrane and similar degrees of proteinuria.

We focused on 6 major podocyte proteins as follows: 3 from the podocyte slit diaphragm: NPHS1, NPHS2, and CD2-associated protein; and 3 from the podocyte cytoskeleton: alpha actinin-4, INF2, and DAG1. Mutations in 5 of these proteins are known to cause FSGS lesions.^18^, ^19^, ^20^, ^21^, ^22^ Although no gFSGS has been found associated with mutations in DAG1 α and β dystroglycan, these molecules are important in the attachment of podocytes to basement membranes; and glomerular expression of dystroglycans has been proposed as able to differentiate MCD from pFSGS.^23^

Our findings show significant loss of all 6 podocyte proteins in MCD, pFSGS, sFSGS, and gFSGS compared with T0 and MN cases. The area examined is approximately the same for all groups, including MCD, FSGS groups, MN, IgAN, and normal controls. We compared the protein of interest to baseline proteins to ensure equal loading, for example, similar to using actin as control in Western blots. If equal amounts were not loaded there would be a wide variation of the spectral counts which is not the case as demonstrated by the very narrow IQR in the MCD and FSGS cases. Our MS findings show that MCD, pFSGS, and sFSGS share a similar pathway of podocyte protein loss that are indistinguishable at MS/MS level. Interestingly, gFSGS showed a relatively lower degree of protein loss, which aligns with its clinical phenotype in adults, which commonly present with nephrotic range proteinuria but no NS. The findings are important in pointing out that in MCD, pFSGS, sFSGS, and gFSGS, even though the injury might be directed against a single protein such as NPHS1, or a secondary cause of podocyte injury, they all culminate in a generalized loss of podocyte proteins of both the slit diaphragm and actin cytoskeleton. Whether this is due to podocyte loss or just podocyte injury is impossible to determine based on our proteomic study, because we did not perform staining for podocyte density. To the best of our knowledge, this is the first study showing widespread loss of podocyte proteins in MCD, pFSGS, sFSGS, and gFSGS. It would be interesting to see if the podocyte proteins reappear in subsequent biopsies following immunosuppression or treatment and remission of the disease, although it is likely that this is the case in patients with MCD and pFSGS who go into complete remission, considering that these patients remain with preserve kidney function long-term.

The podocyte proteomic findings in MN were surprising. Despite the fact that these patients usually present with NS, we found that there was highly variable loss of podocyte proteins in MN. In fact, spectral counts of podocyte-specific proteins in MN biopsies were comparable to those in T0 biopsies and to patients with IgAN. This suggests that the autoimmune process in MN may target proteins of varying biological importance, leading to distinct patterns of injury. Notably, proteins such as NPHS1 and NPHS2—key components of the slit diaphragm—may serve not only as structural anchors but also as central regulators of membrane-associated protein complexes. Their loss may therefore have a more profound impact on podocyte integrity than other proteins.^24^ The findings suggest that the pathophysiology of podocyte injury or effacement in MN is different from that in MCD, pFSGS, and sFSGS. In contrast, cluster analysis shows tight clustering of proteins in MCD, pFSGS, and sFSGS, suggesting that these conditions share a common pathway of podocyte injury and that the extent of podocyte injury, rather than the initiating cause, may be the key determinant of disease progression.

Lastly, the volcano plot analyses show higher spectral counts of other proteins, including apolipoprotein L1, heparan sulfate proteoglycan 2, fibronectin 1, SRGAP2, synaptopodin 2, and catenin alpha 2 in MCD, pFSGS, and sFSGS compared with T0 biopsies. Some of these proteins may play a significant role in the development of sclerosing lesions and further studies are required to determine the role of these proteins in MCD, pFSGS, sFSGS, and gFSGS.

Our study has several limitations. Our cohort is composed of adults and given the high reported incidence of antinephrin antibodies in pediatric versus adult MCD, the proteomic findings may not be fully representative of this subset. We also evaluated a highly selective sample of podocyte proteins, with selection of these mainly based on the possibility of performing IF staining for NPHS1 and NPHS2; and our studies identified complete loss of alpha-actinin-4, INF2, and DAG1, which could not be stained because there was no availability of additional biopsy tissue. However, a recent study on immunohistochemical expression of dystroglycan in biopsies of adults with MCD and FSGS showed no difference in staining patterns between primary and secondary podocytopathies or between steroid-sensitive, -resistant, and -dependent cases of FSGS and MCD.^18^ We could not test for antinephrin antibodies because no serum had been stored. The study did not aim at evaluating potential pathogenetic mechanisms. We did not quantify podocyte numbers by WT1 staining. However, we do not think that our findings could be explained by podocyte depletion in patients with newly diagnosed MCD or pFSGS because significant podocyte depletion has not been demonstrated, at early stages, for either diseases.^25^ We recognize that significant podocyte depletion could be a feature in patients with pFSGS unresponsive to immunosuppression, relapsing disease, or sFSGS because these patients usually have significantly impaired kidney function at presentation. The strength of our study is that we evaluated the IF staining for NPHS1and NPHS2 in all the patients, and IF staining confirmed podocyte loss of NPHS1 and NPHS2. Excluding FSGS of undetermined cause is also a strength of our study, because we have seen cases initially label as “undetermined cause” that with time ended up having genetic FGSG, FSGS due to drugs, etc. Because we wanted to have a clean, well-characterized group of patients in terms of clinical, pathology and genetics, we did not want to contaminate our study with this group of patients.

In conclusion, our study shows that MCD, pFSGS, sFSGS, and gFSGS are indistinguishable by both mass MS/MS, and IF staining demonstrated comparable loss of podocyte proteins (e.g., NPHS1 and NPHS2) across pFSGS, sFSGS, and gFSGS, as well as MCD. These findings support the concept of a common mechanism of injury targeting primarily the podocyte.

## Disclosure

All the authors declared no competing interests.

## Supplementary Material

Supplementary File (PDF) **Figure S1.** Volcano plot MCD versus pFSGS. **Figure S2.** Controls and MN group compared with MCD, pFSGS, and sFSGS. STROBE checklist.
